# Cell-cell contact and matrix adhesion promote αSMA expression during TGFβ1-induced epithelial-myofibroblast transition via Notch and MRTF-A

**DOI:** 10.1038/srep26226

**Published:** 2016-05-19

**Authors:** Joseph W. O’Connor, Krunal Mistry, Dayne Detweiler, Clayton Wang, Esther W. Gomez

**Affiliations:** 1 Department of Chemical Engineering, The Pennsylvania State University, University Park, PA, USA; 2 Department of Biomedical Engineering, The Pennsylvania State University, University Park, PA, USA

## Abstract

During epithelial-mesenchymal transition (EMT) epithelial cells lose cell-cell adhesion, exhibit morphological changes, and upregulate the expression of cytoskeletal proteins. Previous studies have demonstrated that complete disruption of cell-cell contact can promote transforming growth factor (TGF)-β1-induced EMT and the expression of the myofibroblast marker alpha smooth muscle actin (αSMA). Furthermore, increased cell spreading mediates TGFβ1-induced αSMA expression during EMT. Here, we sought to examine how the presence of partial cell-cell contacts impacts EMT. A microfabrication approach was employed to decouple the effects of cell-cell contact and cell-matrix adhesion in TGFβ1-induced EMT. When cell spreading is controlled, the presence of partial cell-cell contacts enhances expression of αSMA. Moreover, cell spreading and intercellular contacts together control the subcellular localization of activated Notch1 and myocardin related transcription factor (MRTF)-A. Knockdown of Notch1 or MRTF-A as well as pharmacological inhibition of these pathways abates the cell-cell contact mediated expression of αSMA. These data suggest that the interplay between cell-matrix adhesion and intercellular adhesion is an important determinant for some aspects of TGFβ1-induced EMT.

Epithelial-mesenchymal transition (EMT) is a process that is of crucial importance in development, carcinogenesis, and organ fibrosis^1^,^2^,^3^. EMT is characterized by loss of epithelial cell apical-basal polarity, downregulation of epithelial markers including E-cadherin, and dissolution of cell-cell junctions. These changes promote an adhesion switch to predominately cell-matrix interactions and are accompanied by drastic morphological changes and the upregulation of a variety of cytoskeletal proteins that contribute to increased cell motility. In addition, studies have demonstrated that a myogenic program can be activated during EMT leading to expression of proteins including alpha smooth muscle actin (αSMA), increased cellular contractility, and acquisition of a myofibroblast phenotype^2^,^4^,^5^,^6^,^7^,^8^.

Transforming growth factor (TGF)-β1, a ubiquitously expressed cytokine, is a potent inducer of EMT. Recent studies have suggested that exposure of epithelial cells to TGFβ1 is not sufficient to induce EMT and that disruption of cell-cell contacts is also necessary for EMT to occur^6^,^9^,^10^. In the presence of TGFβ1, EMT is promoted along the edges of wound sites where cells experience reduced cell-cell contacts^6^,^8^. Breakdown of cell-cell junctions by reduction of calcium levels or downregulation of E-cadherin in combination with treatment with TGFβ1 can also induce EMT in confluent monolayers of tubular kidney epithelial cells^7^,^8^. Moreover, confluent monolayers of epithelial cells are refractive to the EMT inductive signals of TGFβ1 when compared to subconfluent cultures with fewer intercellular contacts^6^,^8^,^11^. With these approaches, modulation of cell-cell contacts can result in variations in cell-matrix interactions or can influence other cellular signaling pathways. For example, cells located along the edges of wound sites can exhibit increased cell spreading in comparison to cells found in interior regions of a monolayer. Additionally, calcium levels affect many cell functions, either directly or indirectly, as calcium plays important roles in maintenance of cell junctional complexes and serves as a second messenger in a wide variety of signal transduction pathways including gene transcription and contraction^12^,^13^,^14^,^15^. As such, it has been challenging to examine the impact of cell-cell contact on EMT in the absence of other factors.

Our recent studies indicate that cell-ECM adhesion and cell spread area are important regulators of the development of myofibroblasts from epithelial cells during TGFβ1-induced EMT^16^. Individual cells (lacking cell-cell contact) that were permitted to spread expressed increased levels of αSMA, a hallmark of the myofibroblast phenotype, and other cytoskeletal associated proteins in response to TGFβ1 treatment while restricting cell spreading blocked TGFβ1-induced expression of myofibroblast markers. Intact cell-cell contacts can limit cell spreading and may therefore impact EMT induction and reduce the expression of αSMA. Furthermore, it is not clear how partial cell-cell contacts (such as those experienced by cells along a wound edge) and cell-ECM adhesion act in concert to mediate the expression of cytoskeletal proteins and myofibroblast development from epithelial cells.

TGFβ1-induced αSMA expression is regulated by the interactions of transcription factors such as CBF1/Suppressor of Hairless/LAG-1 (CSL; also known as RBP-Jκ) and serum response factor (SRF) and their cofactors Notch1 and myocardin-related transcription factor (MRTF)-A, respectively^8^,^17^,^18^. Notch signaling is important for controlling cell fate including smooth muscle cell differentiation^17^,^19^,^20^,^21^ and myofibroblast activation from alveolar epithelial cells^22^ and kidney tubular epithelial cells^23^. Activation of Notch occurs in a cell contact-dependent manner and is initiated when the Notch receptor binds to the transmembrane ligand, Jagged/Delta, on the surface of an adjacent cell. Proteolytic cleavage of membrane bound Notch by γ-secretase releases the Notch intracellular domain (NICD) which can translocate into the nucleus to interact with CSL to promote gene expression. Furthermore, TGFβ1 has been shown to induce the expression of Jagged1 and the Notch-regulated transcriptional repressor Hey1^23^,^24^. MRTF-A also plays an important role in EMT^4^,^7^,^8^,^16^,^25^,^26^,^27^, fibrosis^28^, and metastasis^29^. The subcellular localization and activity of MRTF-A is controlled by the organization of the actin cytoskeleton. We have previously demonstrated that cell-ECM adhesion and matrix rigidity regulate αSMA expression in part by controlling the nuclear accumulation of MRTF-A^4^,^16^. Nevertheless, further studies are needed to elucidate how intercellular contacts and cell-ECM adhesion cues impact these pathways to regulate αSMA expression during EMT.

Here, we sought to determine the interplay between cell-matrix adhesion and cell-cell contacts in TGFβ1-induced EMT. Cells were cultured at varying cell densities and the ability of cells to undergo EMT and express myofibroblast markers was monitored as a function of cell spread area and number of neighboring cells by immunofluorescence staining and western blotting. We demonstrate that induction of EMT by TGFβ1 is controlled by a combination of cell spread area and direct cell-cell interactions. Interestingly, we find that the percentage of cells expressing αSMA increases with both cell spread area and number of neighboring cells. In addition, we identify Notch1 and MRTF-A as important regulators in intercellular adhesion controlled αSMA expression. These data suggest that under certain circumstances cell-cell interactions may in fact promote the expression of myofibroblast markers during TGFβ1-induced EMT.

## Results

### Cell density regulates TGFβ1-induced EMT

**Figure 1:**
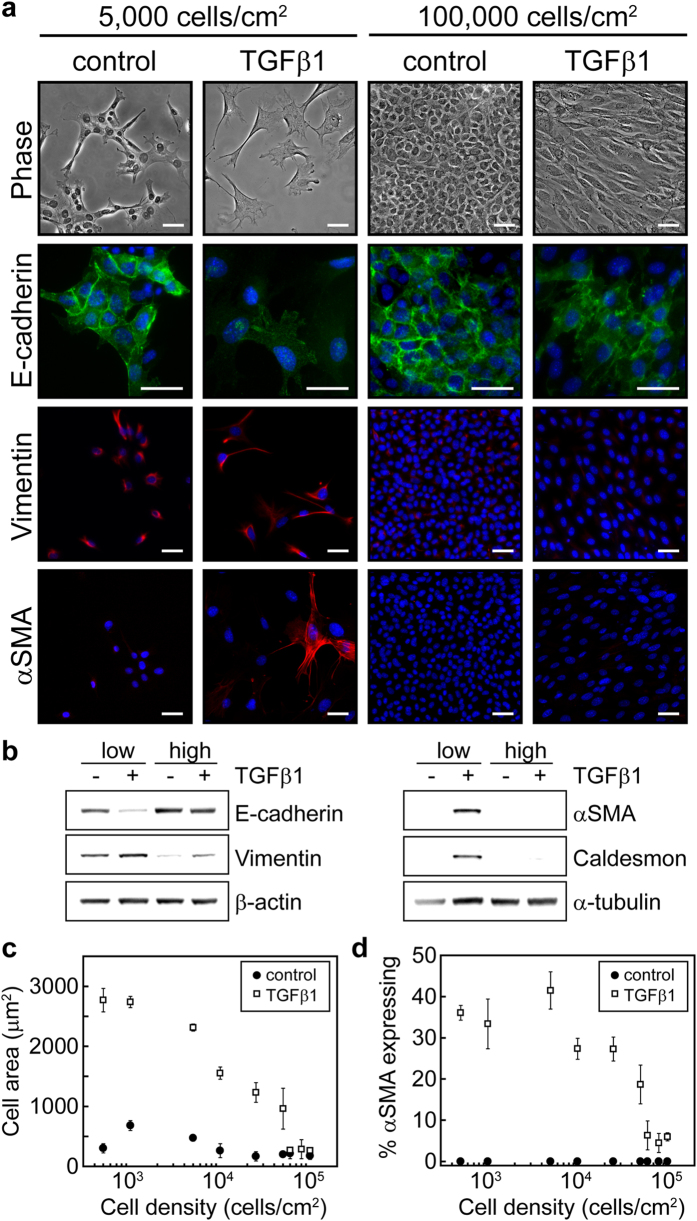
Increasing cell density blocks TGFβ1-induced EMT. (**a**) Phase contrast microscopy images of NMuMG cells and immunofluorescence staining of EMT markers at seeding densities of 5,000 cells/cm^2^ and 100,000 cells/cm^2^ with and without TGFβ1 treatment. Blue stain shows cell nuclei. Scale bars: 50 μm. (**b**) Western blot analysis of EMT markers for cells seeded at low (5,000 cells/cm^2^) and high (100,000 cells/cm^2^) densities with and without TGFβ1. (**c**) Mean cell area as a function of cell seeding density. (**d**) Percentage of cells expressing αSMA as a function of cell seeding density.

To assess the combined contributions of cell-cell contact and cell spreading on TGFβ1-induced EMT the effect of cell density on the expression of EMT markers was investigated. Normal murine mammary gland (NMuMG) epithelial cells were seeded at densities ranging from 500 to 100,000 cells/cm^2^ and were then treated with TGFβ1 or control vehicle for 48 hours. The expression of EMT markers was then analyzed by immunofluorescence staining and by western blotting. Cells plated at high densities were refractive to TGFβ1-induction of EMT while cells cultured at low densities exhibited reduced expression of the epithelial marker E-cadherin and increased expression of the mesenchymal markers vimentin and αSMA (Fig. 1a,b). TGFβ1-treated cells exhibited increased projected cell area in comparison to control cells, especially at low cell densities, and the projected cell area of control and TGFβ1-treated cells decreased with increasing cell density (Fig. 1c). Furthermore, the percentage of cells expressing αSMA decreased with increasing cell density (Fig. 1d). Similar results were obtained for Madine-Darby Canine Kidney (MDCK) epithelial cells (Fig. S1a,b). These data suggest that the interplay between cell-cell contact and cell spreading may regulate aspects of EMT.

### Cell-cell interactions and cell-ECM adhesion together control αSMA expression during TGFβ1-induced EMT

**Figure 2:**
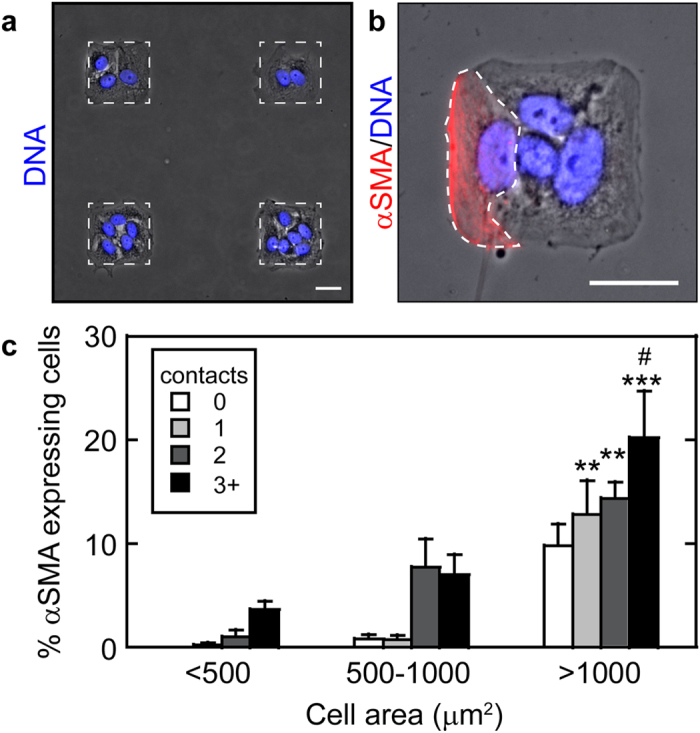
TGFβ1-induced alpha smooth muscle actin expression increases with cell spread area and cell-cell interactions. (**a**) Array of micropatterned NMuMG cells cultured on fibronectin islands. Protein islands are outlined by a white dotted line. (**b**) Square NMuMG epithelial tissue demonstrating cell area, number of contacting neighbors, and αSMA expression. A single cell expressing αSMA is outlined by a white dotted line. Scale bars: 25 μm. (**c**) Percentage of NMuMG cells expressing αSMA as a function of cell area and number of neighboring cells following treatment with TGFβ1. #p < 0.05 compared to >1000 μm^2^, 0 contact. **p < 0.005, ***p < 0.0001 compared to <500 μm^2^, 0 contact.

To further examine the combined effects of cell-cell contact and cell area on TGFβ1-induced expression of αSMA during EMT, cells were plated to micro-contact printed islands of fibronectin ranging in size from 400 μm^2^ to 6,400 μm^2^ in area. By varying the size of the island as well as the number of cells plated to each island, the cell spread area and the amount of cell-cell contact were controlled (Fig. 2a,b). The percentage of cells expressing αSMA increased significantly in cells with zero contacts as cell spread area increased (Fig. 2c). These data are consistent with our previous studies demonstrating that cell spreading is necessary for TGFβ1-induced expression of myofibroblast markers such as αSMA^16^. Furthermore, the percentage of cells expressing αSMA increased with increased cell-cell interactions for each range of cell spread areas that was explored. These findings suggest that cells may respond to neighboring cells to control EMT and that under some circumstances cell-cell interactions may positively regulate αSMA expression during TGFβ1-induced EMT.

**Figure 3:**
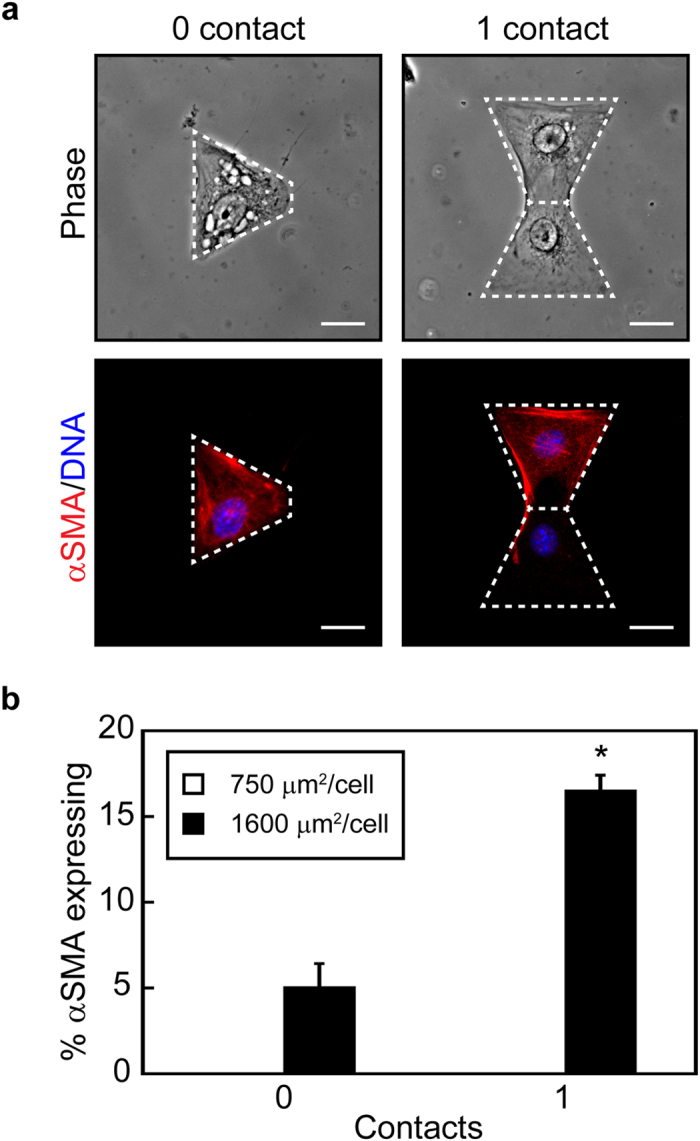
TGFβ1-treated NMuMG epithelial cells in direct contact with other cells express αSMA more readily than single cells when permitted to spread. (**a**) Phase contrast images of cells and fluorescence microscopy images of αSMA expression in NMuMG cells cultured within micropatterned triangular and bowtie arrays. Scale bars: 20 μm. (**b**) Percentage of NMuMG epithelial cells expressing αSMA when cultured with TGFβ1 for cell spread areas of 750 and 1600 μm^2^/cell within micropatterned triangular and bowtie arrays. *p < 0.005 compared to 1600 μm^2^, 0 contact.

To decouple the effects of cell-cell contact and cell-matrix adhesion we utilized a micropatterning technique that enables precise control over cell spread area and the number of neighboring cells^30^. Triangular or bowtie shaped islands of defined size were microcontact printed onto slides and NMuMG cells were seeded at one cell per triangular region of the bowtie (Fig. 3a). Treatment with TGFβ1 resulted in an increase in the percentage of cells expressing αSMA both in single cells and in cell pairs with cell spread areas of 1600 μm^2^/cell, with a significant increase in αSMA expression in cell pairs in comparison to cells lacking neighbors (Fig. 3b). Interestingly, confining cell spread area to 750 μm^2^/cell abrogated the expression of αSMA in both single and doublet cells (Fig. 3b). Cells treated with the vehicle control exhibited no αSMA expression for all culture conditions (data not shown). Similar results were obtained for MDCK cells (Fig. S1c). These data confirm that cell-cell interactions can play an important role in promoting the expression of αSMA during TGFβ1-induced EMT and suggest that a threshold cell spread area may be necessary for cell-cell interaction induced αSMA.

### Direct cell-cell contact mediates αSMA expression during TGFβ1-induced EMT

**Figure 4:**
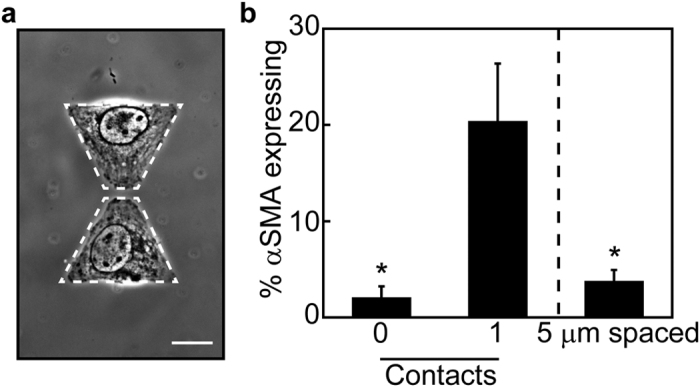
Paracrine signaling is not responsible for increased αSMA expression. (**a**) Phase contrast image of NMuMG cells cultured on 1600 μm^2^/cell triangular islands separated by 5 μm. Scale bar: 20 μm. (**b**) Percentage of single (0 contact), doublet (1 contact), and 5 μm separated NMuMG cells expressing αSMA with TGFβ1 treatment. *p < 0.05 compared to doublet (1 contact) cells.

The effects of cell-cell interactions on TGFβ1-induced αSMA expression could possibly be promoted by a soluble factor secreted by cells or by signaling initiated by direct cell-cell contact between neighboring cells. To examine the role of paracrine signaling in cell-cell interaction promoted αSMA expression during TGFβ1-induced EMT, bowtie shaped features were fabricated in which the triangular regions (1600 μm^2^) were separated by 5 μm (Fig. 4a). When cultured on separated features, the percentage of cells expressing αSMA in response to TGFβ1 decreased to single cell levels (Fig. 4b). This data suggests that direct contact between cells is responsible for the increase in the expression of αSMA in response to TGFβ1 observed in cell pairs.

### Intercellular contacts and cell-ECM adhesion control αSMA expression through regulating activation of Notch1

**Figure 5:**
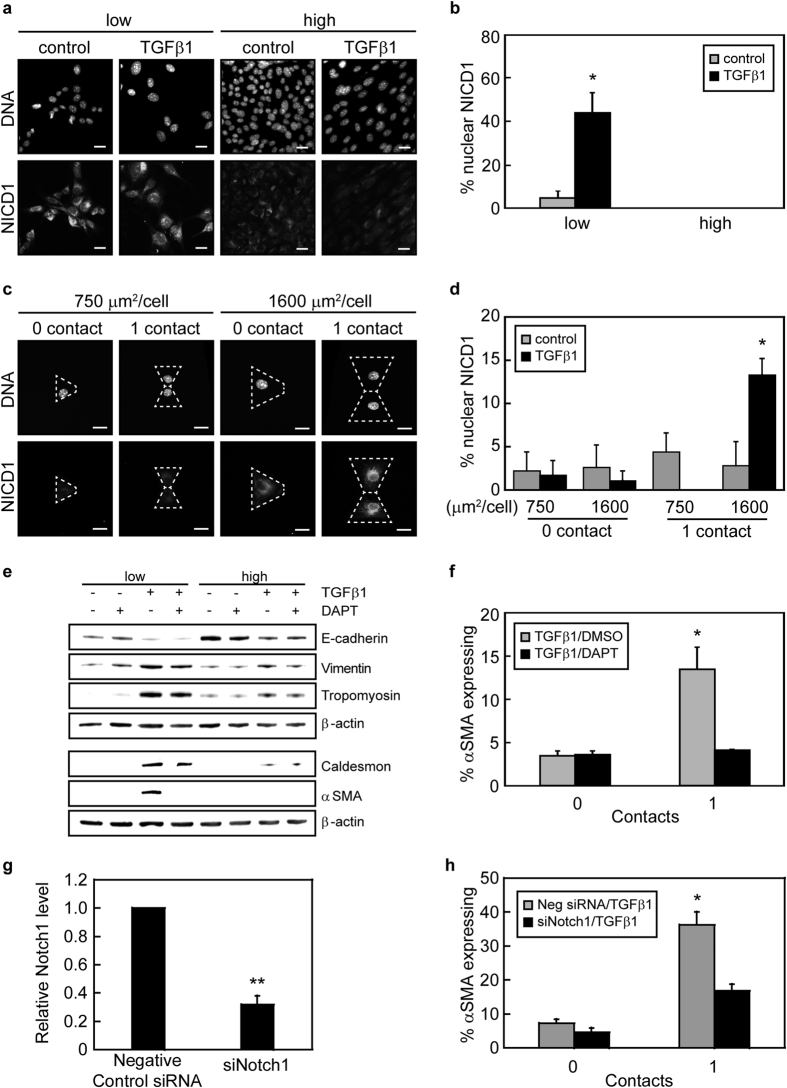
Cell-cell contact and cell-matrix adhesion together regulate αSMA expression via the Notch1 signaling pathway. (**a**) Fluorescence microscopy images of NICD1 localization in cells seeded at low and high densities with and without TGFβ1. Scale bars: 20 μm. (**b**) Quantification of the percentage of cells with nuclear NICD1 as a function of seeding density. *p < 0.05 compared to all samples. (**c**) Fluorescence microscopy images of NICD1 localization in TGFβ1-treated NMuMG cells cultured on micropatterned triangular and bowtie shaped islands. Dotted white lines outline an individual cell. Scale bars: 20 μm. (**d**) Quantification of the percentage of cells with nuclear NICD1 as a function of cell spread area and number of neighboring cells. *p < 0.05 compared to all samples. (**e**) Western blot analysis of EMT markers for cells seeded at low (5,000 cells/cm^2^) and high (100,000 cells/cm^2^) densities with and without TGFβ1 and DMSO control vehicle or γ-secretase inhibitor DAPT. (**f**) Percentage of NMuMG cells with a cell spread area of 1600 μm^2^ expressing αSMA on triangular (0 contact) and bowtie (1 contact) islands following treatment with TGFβ1 and DMSO control vehicle or γ-secretase inhibitor DAPT. *p < 0.05 compared to all samples. (**g**) Transcript levels of Notch1 for cells transfected with siRNA. **p < 0.01 compared to negative control siRNA. (**h**) Percentage of NMuMG cells transfected with siRNA targeting Notch1 with a cell spread area of 1600 μm^2^ expressing αSMA on triangular (0 contact) and bowtie (1 contact) islands following treatment with TGFβ1. *p < 0.05 compared to all samples.

Notch1 signaling is upregulated during EMT and has been linked to αSMA expression^17^,^22^. Furthermore, the activation of Notch1 is dependent on intercellular contacts; thus, we hypothesized that Notch1 signaling might be involved in the cell-cell contact and cell-ECM adhesion control of TGFβ1-mediated αSMA expression. Western blotting revealed that treatment of NMuMG cells with TGFβ1 induced the expression of Jagged1, a Notch1 ligand, equally in cells cultured at both low and high densities (Fig. S2). Following ligand engagement, the Notch1 intracellular domain (NICD1) is cleaved and can then localize to the cell nucleus to promote gene expression. Examination of the localization of NICD1 by immunofluorescence staining showed that NICD1 was nuclearly localized in approximately 40% of epithelial cells when cultured at low cell densities and treated with TGFβ1, whereas NICD1 infrequently localized to the nucleus within cells cultured at high densities (Fig. 5a,b). Nuclear localization of NICD1 in cells cultured at low densities with TGFβ1 treatment correlated with an increased percentage of cells exhibiting αSMA expression (Fig. 1c,d and Fig. S3). Moreover, NICD1 was also observed to localize within the nucleus of TGFβ1-treated cell pairs with a spread area of 1600 μm^2^/cell and lack of cell-cell contact reduced the percentage of cells exhibiting nuclear NICD1 (Fig. 5c,d). Interestingly, confinement of cell spread area to 750 μm^2^/cell also blocked nuclear localization of NICD1 in cell pairs. These data suggest that both cell-cell contact and cell spreading are important regulators of Notch1 activation during TGFβ1-induced EMT.

To examine whether Notch activation is necessary for the cell-cell contact mediated increase in αSMA expression cells were treated with DAPT, a γ-secretase inhibitor that blocks cleavage of the Notch intracellular domain. Western blotting showed that treating cells with DAPT decreased the expression of αSMA in TGFβ1-treated NMuMG cells cultured at low densities however the expression level of the epithelial marker E-cadherin was not impacted (Fig. 5e). Furthermore, immunofluorescence staining revealed that the percentage of cells expressing αSMA in TGFβ1-treated cell pairs with a cell spread area of 1600 μm^2^/cell significantly decreased with DAPT treatment (Fig. 5f). Similar results were obtained for MDCK cells (Fig. S1d). To determine whether Notch1 is necessary for the cell-cell contact mediated increase in αSMA expression during TGFβ1-induced EMT, siRNA was used to knockdown Notch1 expression (Fig. 5g). Immunofluorescence staining of siRNA transfected cells revealed that the percentage of cells expressing αSMA in TGFβ1-treated cell pairs with a cell spread area of 1600 μm^2^/cell significantly decreased in Notch1 knockdown cells in comparison to negative control siRNA treated cells (Fig. 5h). Together, these data suggest that the combined effects of cell-cell contact and cell spread area control TGFβ1-induced expression of αSMA through the Notch1 signaling pathway.

### Intercellular contacts and cell-ECM adhesion control αSMA expression through regulating the subcellular localization of MRTF-A

**Figure 6:**
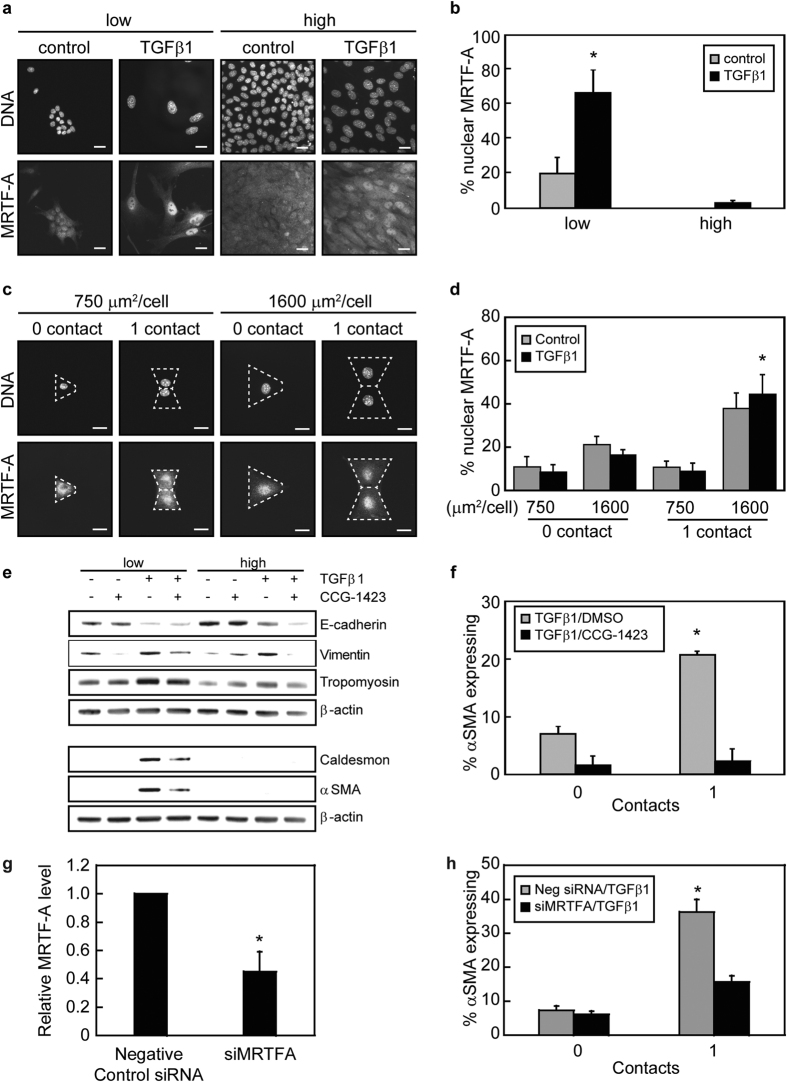
Cell-cell contact and cell-matrix adhesion together regulate αSMA expression by controlling MRTF-A subcellular localization. (**a**) Fluorescence microscopy images of MRTF-A localization in cells seeded at low and high densities with and without TGFβ1. Scale bars: 20 μm. (**b**) Quantification of the percentage of cells with nuclear MRTF-A as a function of cell seeding density. *p < 0.05 compared to all samples. (**c**) Fluorescence microscopy images of MRTF-A localization in TGFβ1-treated cells cultured on micropatterned triangular and bowtie shaped islands. Dotted white lines outline an individual cell. Scale bars: 20 μm. (**d**) Quantification of the percentage of cells with nuclear MRTF-A as a function of cell spread area and number of neighboring cells. *p < 0.05 compared to 1600 μm^2^, 0 contact, TGFβ1. (**e**) Western blot analysis of EMT markers for NMuMG cells seeded at low (5,000 cells/cm^2^) and high (100,000 cells/cm^2^) densities with and without TGFβ1 and DMSO control vehicle or CCG-1423. (**f**) Percentage of NMuMG cells with a cell spread area of 1600 μm^2^ expressing αSMA on triangular (0 contact) and bowtie (1 contact) islands following treatment with TGFβ1 and DMSO control vehicle or CCG-1423. *p < 0.05 compared to all samples. (**g**) Transcript levels of MRTF-A for cells transfected with siRNA. *p < 0.05 compared to negative control siRNA. (**h**) Percentage of NMuMG cells transfected with siRNA targeting MRTF-A with a cell spread area of 1600 μm^2^ expressing αSMA on triangular (0 contact) and bowtie (1 contact) islands following treatment with TGFβ1. *p < 0.05 compared to all samples.

MRTF-A/SRF complexes bind to elements within the promoters of cytoskeletal associated genes including αSMA^25^,^27^. Previous studies have demonstrated that disruption of cell-cell contacts is important for initiation of TGFβ1-induced EMT and MRTF-A mediated transcription^8^,^31^,^32^. Thus, we sought to determine the impact of cell density on MRTF-A subcellular localization during TGFβ1-induced EMT in mammary epithelial cells. Culturing NMuMG cells at low and high densities revealed that MRTF-A localized to the nucleus in a greater percentage of cells cultured at low densities than at high densities (Fig. 6a,b). Furthermore, TGFβ1 treatment of cells cultured at low densities resulted in an increase in cell spread area and an increase in MRTF-A nuclear localization in comparison to control vehicle treated cells. Conversely, when cell area was constricted by cell crowding at the higher densities, only a small population of cells treated with TGFβ1 exhibited nuclear localization of MRTF-A (Fig. 6a,b). These data suggest that cell-cell contact can impact MRTF-A subcellular localization and are consistent with previously published studies demonstrating disruption of intercellular adhesions regulates MRTF-A.

Our previous studies have shown that an increase in cell spread area promotes localization of MRTF-A to the cell nucleus which impacts TGFβ1-induced expression of αSMA^16^. In light of these findings, we next sought to determine the impact of cell-cell contacts on MRTF-A subcellular localization when the cell adhesive area is precisely controlled. Single cells cultured on micropatterned 1600 μm^2^/cell triangular islands contained slightly greater MRTF-A nuclear localization compared to cells cultured on 750 μm^2^/cell islands (Fig. 6c,d). Interestingly, the percentage of cells displaying nuclear localized MRTF-A significantly increased for cell pairs with cell spread areas of 1600 μm^2^/cell, while direct cell-cell contact did not impact MRTF-A localization in cells confined to a cell spread area to 750 μm^2^/cell (Fig. 6c,d). These data suggest that MRTF-A nuclear localization is controlled by the combined effects of cell spread area and cell-cell contact.

To determine whether MRTF-A nuclear localization was necessary for the cell-cell contact mediated increase in αSMA expression, cells were treated with CCG-1423, an inhibitor of MRTF-A nuclear import^33^,^34^. Western blotting revealed that treatment with CCG-1423 reduced the expression of αSMA and caldesmon in cells cultured at low cell densities (Fig. 6e). However, treatment with CCG-1423 did not restore E-cadherin protein levels at low or high cell plating densities. CCG-1423 treatment also significantly reduced the percentage of 1600 μm^2^/cell pairs expressing αSMA (Fig. 6f). Similar results were obtained for MDCK cells (Fig. S1e). To determine whether MRTF-A expression is necessary for the cell-cell contact mediated increase in αSMA expression during TGFβ1-induced EMT, siRNA was used to knockdown MRTF-A expression (Fig. 6g). Immunofluorescence staining of siRNA transfected cells revealed that the percentage of cells expressing αSMA in TGFβ1-treated cell pairs with a cell spread area of 1600 μm^2^/cell significantly decreased in MRTF-A knockdown cells in comparison to control siRNA treated cells (Fig. 6h). Together, these data suggest that MRTF-A mediates the effects of cell-cell contact on αSMA expression during TGFβ1-induced EMT.

## Discussion

In this study, we found that the combined effects of cell-cell contact and cell-matrix adhesion regulate TGFβ1-induced EMT. Culture of epithelial cells at low cell densities, where individual cells lack neighbors and are permitted to spread, promoted decreased expression of epithelial markers and increased expression of mesenchymal markers. In contrast, culture of cells at high densities with intact cell-cell contacts blocked cell spreading and abrogated cell responsiveness to EMT inductive cues. Through the use of a microfabricated model system in which cell-cell contact and cell-matrix adhesion can be precisely controlled and decoupled, we demonstrated that the presence of partial cell-cell contacts can promote TGFβ1-induced expression of the myofibroblast marker αSMA. For cells that are treated with TGFβ1 and that are permitted to spread, cell-cell contact increased the expression of αSMA. Conversely, restricting cell spreading blocked TGFβ1-mediated upregulation of αSMA.

A two-hit model for EMT has been proposed in which both disruption of intercellular contacts and TGFβ1 signaling are indispensable for αSMA expression and myofibroblast development^7^,^35^,^36^. Intact and injured regions of the epithelium respond differentially to TGFβ1 treatment, with cells located along a wound edge exhibiting increased sensitivity to TGFβ1 in comparison to cells found in the interior region of intact epithelial monolayers^36^. Cells located along wound edges that exhibit partial cell-cell contacts can potentially increase in cell spread area in response to TGFβ1 treatment. Thus, in this context partial cell-cell contacts may serve to promote αSMA expression and myofibroblast development. Furthermore, reduction of calcium levels or E-cadherin downregulation results in complete loss of cell-cell contacts in epithelial monolayers. While these treatments alone do not induce αSMA expression, when combined with TGFβ1 treatment αSMA transcript and protein levels increase^6^,^8^. Interestingly, these treatments are not expected to result in an increase in cell spread area, but rather a complete disruption of cell-cell contacts. As such, the degree of cell-cell contact (partial or total loss of contact) may be important for fine tuning the regulation of TGFβ1-induced αSMA expression.

Myofibroblast development from epithelial cells is mediated by Smad3 and MRTF-A, with cell-cell contact disassembly promoting MRTF-A nuclear localization and αSMA transcription^7^. Our results are consistent with this mechanism as we find that epithelial cells cultured at high cell densities are refractive to EMT inductive cues (Fig. 1). In addition, subconfluent cultures (with few or no cell-cell contacts) exhibited TGFβ1-induced MRTF-A nuclear localization and changes in EMT markers. However, our results suggest that disruption of cell-cell contacts is not sufficient to promote the expression of αSMA during TGFβ1-induced EMT as individual cells cultured under conditions that blocked cell spreading (750 μm^2^/cell) did not express αSMA. These findings are consistent with prior results from our group^16^. Interestingly, our data also demonstrates that when cell spread area is accounted for and cells are permitted to spread (1600 μm^2^/cell), cell-cell contacts in fact promote the expression of αSMA (Fig. 3).

The studies presented here suggest that cell-cell interaction mediated expression of αSMA during TGFβ1-induced EMT results from direct cell-cell contact rather than a secreted factor. The Notch signaling pathway is directed by intercellular contacts and can regulate TGFβ1-induced EMT^22^,^23^. We find that Notch1 is necessary for the increase in αSMA expression due to direct cell-cell contact, as knockdown of Notch1 expression or inhibition of Notch activation abrogated the cell-cell contact mediated expression of αSMA when cells were permitted to spread. Cells cultured at both low and high cell densities express equal levels of Jagged1 in response to TGFβ1 treatment suggesting that the Notch ligand is equally available for engagement with Notch in both low and high density culture conditions. Following Notch/Jagged binding, the Notch intracellular domain is then proteolytically cleaved by γ-secretase which releases it to translocate into the nucleus to interact with CSL and to direct the expression of αSMA^22^,^37^. We find that NICD1 activation and localization to the cell nucleus occurs when cells are permitted to spread, have intercellular contacts with neighbors, and are treated with TGFβ1. While these findings suggest that the Notch pathway is regulated by a combination of cell spreading and cell-cell contact, further studies are necessary to elucidate mechanistically how cell spread area impacts Notch signaling.

The TGFβ and Notch signaling pathways can interact in a variety of ways. For example, TGFβ1 induces the expression of the Notch ligand, Jagged1, and the Notch-regulated transcriptional repressor Hey1^23^,^24^. Furthermore, the TGFβ and Notch pathways also intersect through Smad3, which facilitates nuclear import of Notch1^38^ and assists in the activation of promoters with Notch1 and CSL^24^. Interestingly, Smad3 is also an inhibitor of MRTF-A signaling and αSMA expression^7^,^35^. The transcriptional activity of MRTF-A is blocked when bound to Smad3 and degradation of Smad3 during later stages of TGFβ1-induced EMT frees MRTF-A to associate with SRF and to bind to CArG boxes of the αSMA promoter^7^,^9^. It is possible that the recruitment of Smad3 by Notch1 can regulate the activity of MRTF-A to introduce increased expression levels of αSMA. In addition, Notch activation promotes myosin light chain phosphorylation and RhoA activation in endothelial cells^39^ and is upstream of cytoskeletal rearrangements during TGFβ1-induced EMT in keratinocytes^23^ and human kidney epithelial cells^40^. Because MRTF-A subcellular localization is sensitive to the levels of monomeric and filamentous actin^41^,^42^, Notch activation may also regulate MRTF-A signaling through control of cytoskeletal rearrangements during EMT. Here, we find that the relative levels of filamentous actin and phosphorylated myosin increase with an increase in cell spread area, but are not significantly impacted by cell-cell contact (Fig. S4). Furthermore, inhibition of Notch activation by treatment of cells with DAPT does not block the subcellular localization of MRTF-A during TGFβ1-induced EMT in cells with fixed spread areas (Fig. S5). Likewise, blocking MRTF-A nuclear import does not impact NICD1 subcellular localization (Fig. S6). These findings suggest that Notch activation likely does not regulate MRTF-A signaling through control of the cytoskeleton in this system. Accordingly, further molecular investigations are required to determine whether there is interplay between Notch and MRTF-A pathways in the regulation of TGFβ1-induced αSMA expression.

Our findings demonstrate that the combined effects of cell-ECM adhesion, cell-cell contacts, and TGFβ1 signaling are important regulators of activated Notch1, MRTF-A subcellular localization, and αSMA expression during TGFβ1-induced EMT. These data suggest that the partial cell-cell contacts that epithelial cells experience along a wound site may serve to promote TGFβ-induced αSMA expression and myofibroblast development. As such, targeting cell adhesions, Notch1, or MRTF-A may be useful for preventing myofibroblast development from epithelial cells under pathological conditions or for promoting myofibroblast development along injured epithelium to aid in wound healing.

## Methods

### Cell culture and reagents

Normal murine mammary gland (NMuMG) epithelial cells were obtained from American Type Culture Collection and maintained in Dulbecco’s Modified Eagle Medium (DMEM) supplemented with 10% fetal bovine serum (FBS; Atlanta Biologicals), 10 μg/ml insulin (Sigma), and 50 μg/ml gentamicin (Life Technologies). Madine-Darby canine kidney (MDCK) epithelial cells were obtained from Celeste Nelson (Princeton University) and maintained in Eagle’s Minimum Essential Medium (MEM) supplemented with 10% FBS and 50 μg/ml gentamicin. Cells were cultured in complete growth media in a humidified incubator at 37 °C with 5% CO_2_. Cells were serum starved for 12 hours prior to treating with 10 ng/ml of recombinant human TGFβ1 (R&D Systems) or carrier solution for 48 hours. For inhibitor studies, cells were treated with the following reagents diluted in dimethyl sulfoxide (DMSO): DAPT (10 μM, Sigma); or CCG-1423 (7.5 μM, Enzo).

### Patterning substrata by microcontact printing

Micro-contact printing was used to stamp islands of fibronectin onto polydimethylsiloxane (PDMS; Dow Corning) coated glass slides. Master silicon wafers were patterned by standard photolithography techniques and used to cast PDMS template stamps^43^,^44^. Featureless PDMS stamps were coated with 25 μg/ml human fibronectin (BD Biosciences) for 2 h, rinsed thoroughly with 1× phosphate buffered saline (PBS) and were dried with a stream of nitrogen. The template stamps were UV-oxidized for 7 minutes and brought in conformal contact with the featureless stamps to remove fibronectin. The stamps were then brought into conformal contact with PDMS-coated glass coverslips to transfer fibronectin islands of defined shape and size to the coverslip surface. Coverslips were then incubated with a solution of 1% Pluronics F127 (Sigma) to passivate regions not stamped with protein in order to prevent cells from adhering to these regions of the coverslip surface. Following rinsing with 1× PBS, cells were plated in cell culture media to the micropatterned coverslips. After 30 min, samples were rinsed to remove non-adherent cells.

### siRNA transfections

siRNA targeting Notch1 (155626), MRTF-A (170371), and Silencer Negative Control No. 1 siRNA (AM4611) were obtained from Life Technologies. Cells were transfected with siRNA using Lipofectamine RNAiMAX Transfection Reagent (Life Technologies) following the manufacturer’s suggested protocol.

### Quantitative real-time PCR

Total RNA was isolated from cells using a RNeasy Plus kit (Qiagen). cDNA was then synthesized using a High Capacity cDNA Reverse Transcription Kit (Applied Biosystems) and transcript levels were measured on an Applied Biosystems 7300 Real-time PCR system using the following Taqman assays (Life Technologies): Notch1 (Mm00627185_m1); MRTF-A (Mm00461840_m1). mRNA expression was normalized to the expression of the housekeeping gene cyclophilin. Melt curve analysis was performed to verify that a single PCR product was obtained for each sample.

### Immunofluorescence staining

For staining of cytoskeletal associated proteins and NICD1, cells were fixed with an ice-cold solution of 1:1 methanol/acetone at −20 °C for 10 min. For all other proteins, cells were fixed with 4% paraformaldehyde for 15 min at room temperature. Following fixation, cells were rinsed thoroughly with 1× PBS, permeabilized with 0.1% Triton X-100, blocked with 10% goat serum (Sigma), and incubated with the following primary antibodies: αSMA (1A4, Sigma); E-cadherin (Cell Signaling); vimentin (VIM-13.2, Sigma); p-myosin (Cell Signaling); NICD1 (Abcam); or MRTF-A (H140, Santa Cruz Biotechnology). Cells were then incubated with AlexaFluor tagged secondary antibodies (Life Technologies) followed by counterstaining of the cell nuclei with Hoechst 33342 (Life Technologies). Filamentous actin was stained using fluorescently-tagged phalloidin (Life Technologies) following the manufacturer’s suggested protocol. Before imaging, samples were mounted to cover slides using Fluoromount-G (Electron Microscopy Sciences).

### Western blotting

Cells were lysed in a cold RIPA buffer (Thermo Scientific) containing Halt protease and phosphatase inhibitor cocktails (Thermo Scientific). Protein concentrations were determined using a Pierce BCA Protein Assay Kit (Thermo Scientific). Equal amounts of cell lysates were then separated on a NuPAGE Novex 4–12% Bis-Tris gel (Life Technologies) and transferred to PVDF membranes using a Transblot Semi-Dry Transfer Cell (Biorad). Membranes were blocked with a 5% non-fat dry milk solution and then examined with primary antibodies against αSMA (1:2500, Sigma), caldesmon (1:10000, Abcam), vimentin (1:500, Sigma), E-cadherin (1:1000, Cell Signaling), tropomyosin (1:1000, Sigma) α-tubulin (1:1000, Sigma), and β-Actin (1:1000, Cell Signaling). Blots were imaged on a FluorChem FC2 system (Cell Biosciences) through horseradish peroxidase (HRP)-conjugated secondary antibodies (1:2000, Cell Signaling) and SuperSignal West Pico Chemiluminescent Substrate (Pierce).

### Microscopy and analysis

Samples were imaged using a 20× or 40× air objective on a Nikon Eclipse Ti-E inverted fluorescence microscope equipped with a Photometrics CoolSNAP HQ^2^ CCD camera. Cell projected area, the number of neighboring cells, and protein expression were examined using ImageJ software. Cell borders were traced within phase contrast images and the number of neighboring cells was determine based on the number of adjacent cells. The percentage of cells expressing αSMA was computed by determining the number of cells expressing αSMA and dividing by the total number of cells examined. In order to determine the subcellular localization of NICD1 and MRTF-A, the nuclear fluorescence intensities were compared to the cytoplasmic fluorescence intensities within cells. Cells with NICD1 or MRTF-A nuclear fluorescence intensities two-fold greater than cytoplasmic fluorescence intensities were classified as nuclear localized.

All experiments were performed a minimum of three times unless noted and data shown is mean ± standard error of the mean. Statistical analysis was performed using either a two-tailed student’s t-test or analysis of variance (ANOVA) followed by the Bonferroni post-hoc correction using Kaleidagraph v2.4 software. Differences between experimental conditions were considered significant for p < 0.05.

## Additional Information

**How to cite this article**: O’Connor, J. W. *et al*. Cell-cell contact and matrix adhesion promote αSMA expression during TGFβ1-induced epithelial-myofibroblast transition via Notch and MRTF-A. *Sci. Rep.* **6**, 26226; doi: 10.1038/srep26226 (2016).

## Supplementary Material

Supplementary Information
