# Supplementary material for: Cell-cell contact and matrix adhesion promote αSMA expression during TGFβ1-induced epithelial-myofibroblast transition via Notch and MRTF-A
Source: Sci Rep. 2016 May 19;6:26226. doi: 10.1038/srep26226 (PMC4872162; doi:10.1038/srep26226)
Supplement: Supplementary Information [file srep26226-s1.pdf]

Supplementary Information

Cell-cell contact and matrix adhesion promote αSMA expression during TGFβ1-induced epithelial-myofibroblast transition via Notch1 and MRTF-A

Joseph W. O'Connor[1], Krunal Mistry[2], Dayne Detweiler[1], Clayton Wang[1], Esther W. Gomez[1,2,*]

[1]Department of Chemical Engineering, The Pennsylvania State University, University Park, PA

[2]Department of Biomedical Engineering, The Pennsylvania State University, University Park, PA

***corresponding author:**

Esther W. Gomez
The Pennsylvania State University
Department of Chemical Engineering
204 Fenske Laboratory
University Park, PA 16802
e-mail: ewgomez@engr.psu.edu
phone: 814-867-4732

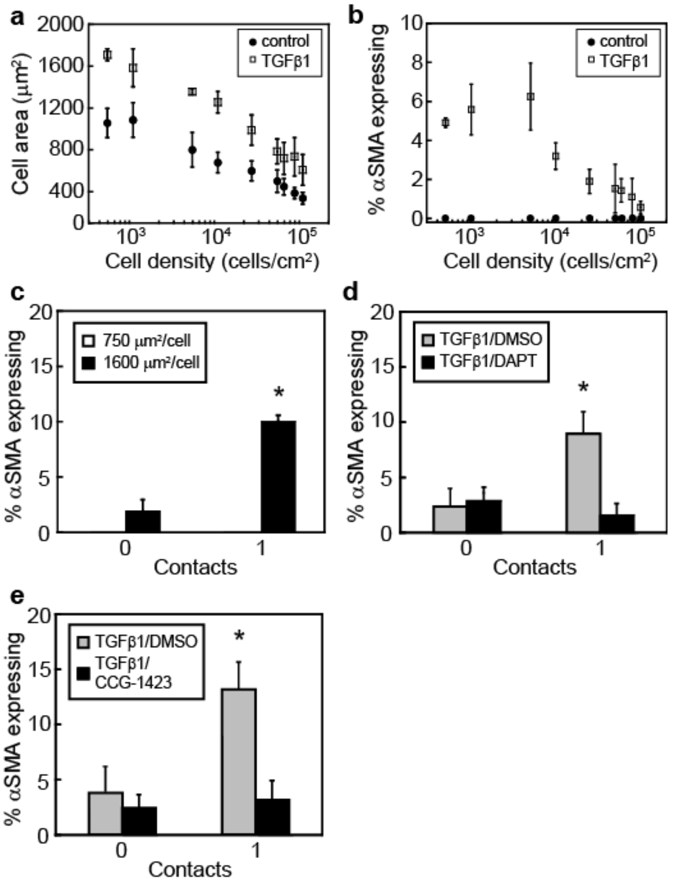

Figure S1. Cell adhesive cues promote TGFβ1-induced αSMA expression in Madine Darby Canine Kidney (MDCK) epithelial cells. (a) Mean cell area as a function of cell seeding density. (b) Percentage of MDCK cells expressing αSMA as a function of cell seeding density. (c) Percentage of single and bowtie TGFβ1-treated MDCK cells expressing αSMA for cell spread areas of 750 and 1600 μm^2/cell. *$p < 0.05$ compared to 1600 μm^2, 0 contact. αSMA expression was not observed for cells cultured at 750 μm^2/cell. (d) Percentage of single and bowtie MDCK epithelial cells expressing αSMA when cultured with TGFβ1 and DMSO or DAPT for a spread area of 1600 μm^2/cell. *$p < 0.05$ compared to all samples. (e) Percentage of single and bowtie MDCK cells expressing αSMA when cultured with TGFβ1 and DMSO or CCG-1423 for a spread area of 1600 μm^2/cell. *$p < 0.05$ compared to all samples.

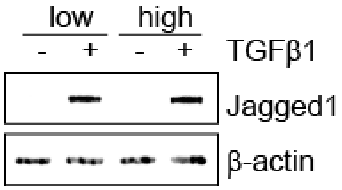

Figure S2. TGFβ1 induces the expression of the Notch ligand Jagged1 in NMuMG cells cultured at low (5,000 cells/cm^2) and high (100,000 cells/cm^2) densities.

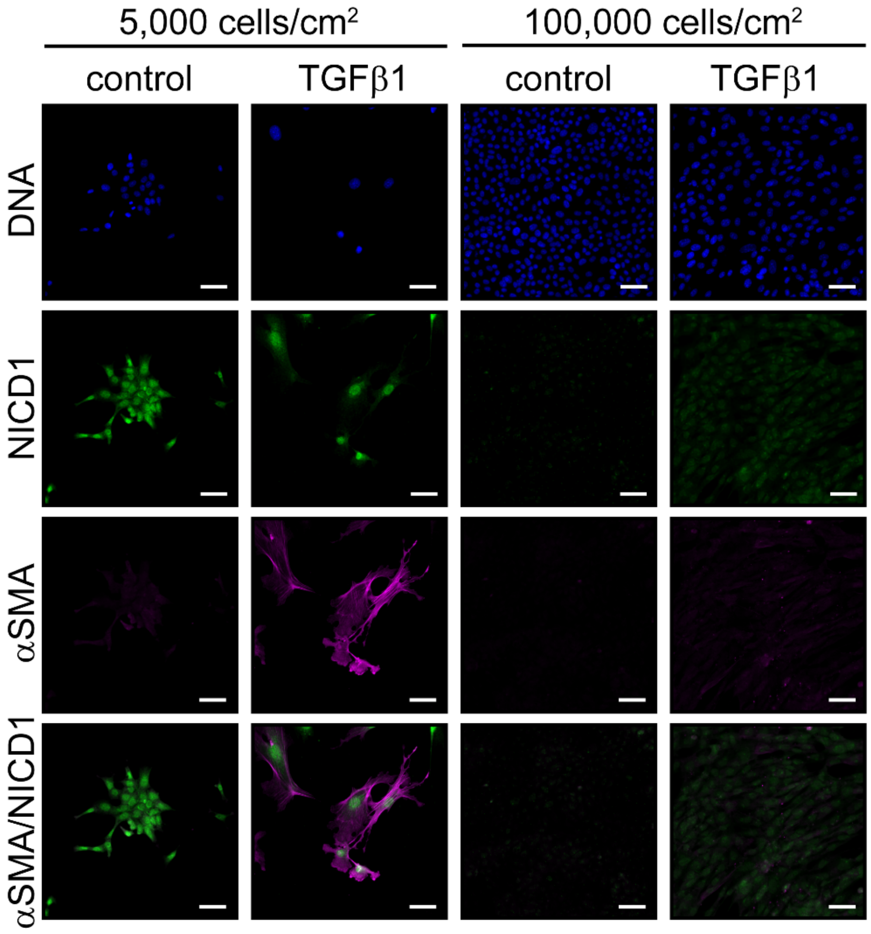

Figure S3. Co-immunostaining for NICD1 and αSMA in NMuMG cells cultured at low (5,000 cells/cm^2) and high (100,000 cells/cm^2) densities. Scale bars: 50 μm.

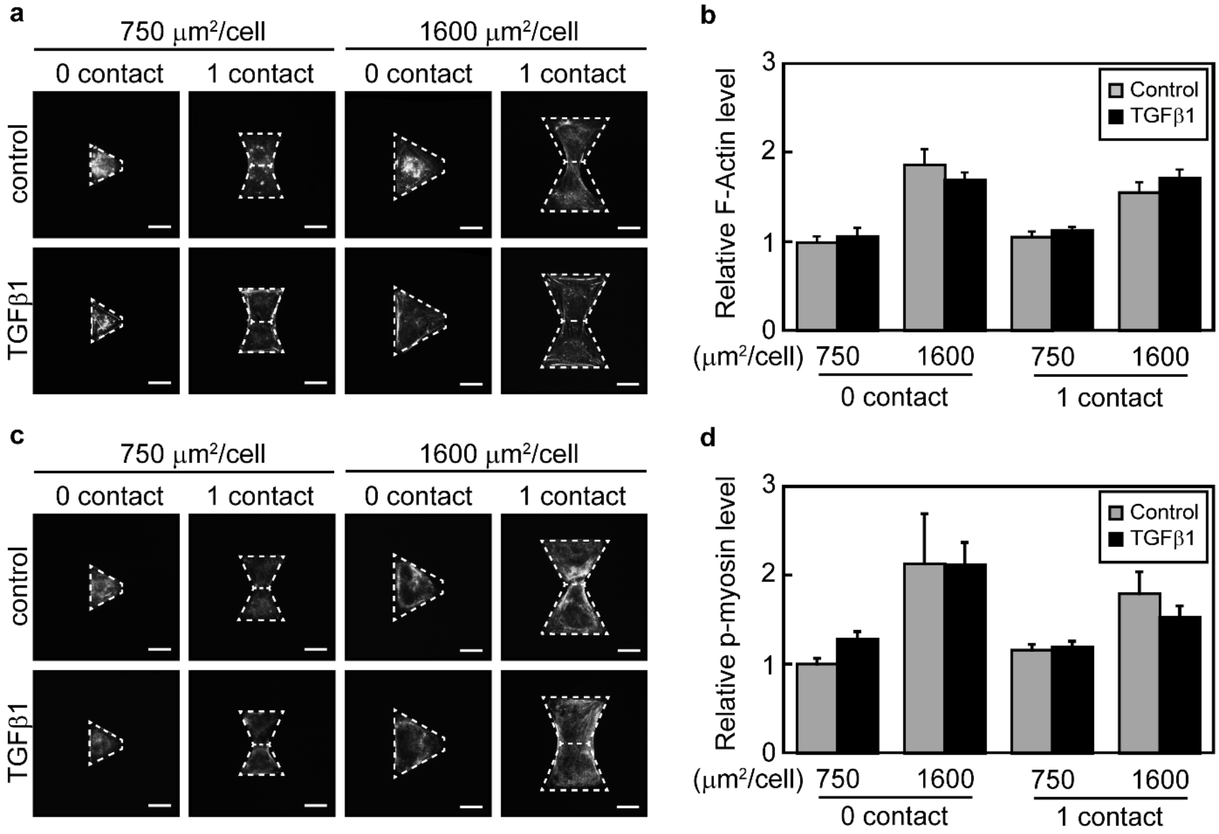

Figure S4. F-actin levels and phosphorylation of p-myosin are dominated by cell spread area. (a) Fluorescence microscopy images of F-actin in cells cultured on 750 μm^2 and 1600 μm^2 per cell islands with zero or one neighboring cell. Scale bars: 20 μm. (b) Quantification of the relative total integrated intensity of phalloidin in NMuMG cells as a function of cell spread area and number of neighboring cells. (n = 2) (c) Fluorescence microscopy images of p-myosin in cells cultured on 750 μm^2 and 1600 μm^2 per cell islands with zero or one neighboring cell. Scale bars: 20 μm. (d) Quantification of the relative total integrated intensity of p-myosin in NMuMG cells as a function of cell spread area and number of neighboring cells. (n = 2)

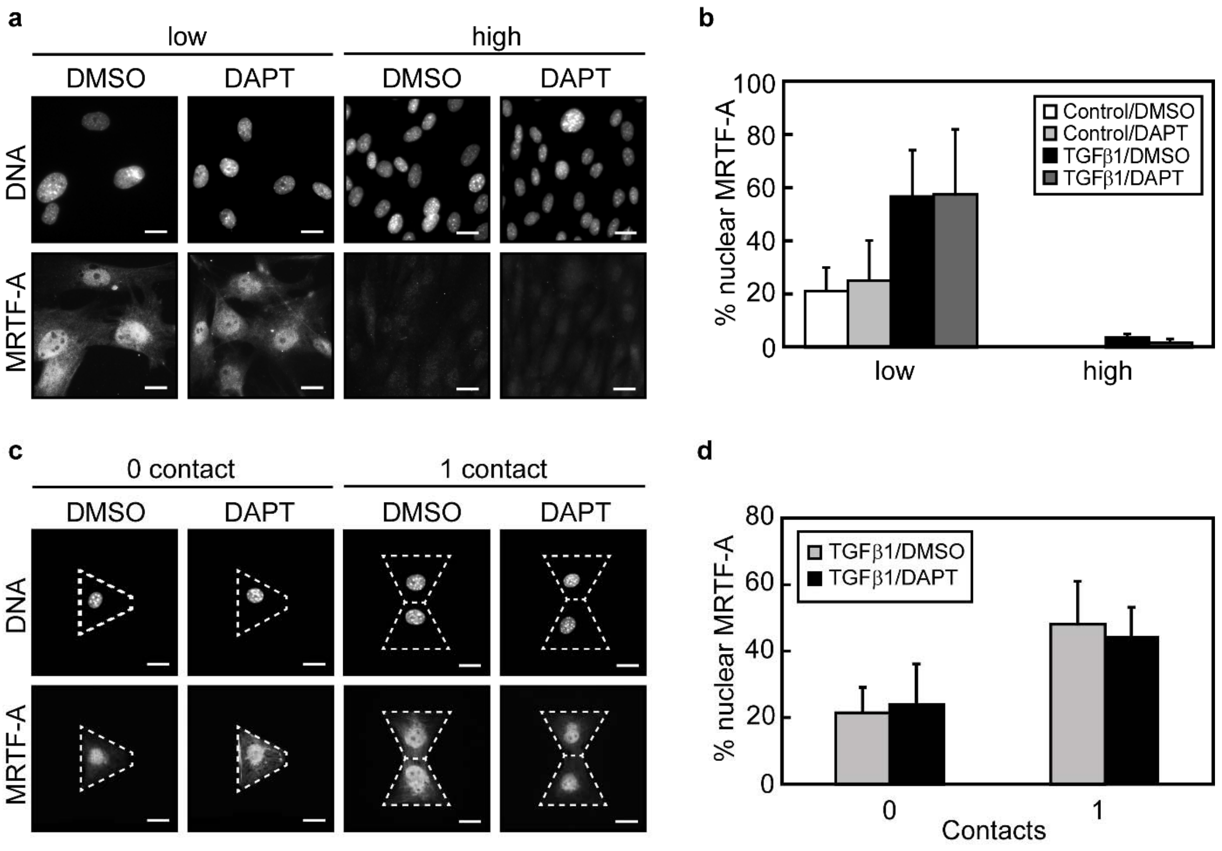

Figure S5. Inhibition of Notch1 activation does not block MRTF-A nuclear localization. (a) Fluorescence microscopy images of MRTF-A localization in NMuMG cells seeded at low and high densities and treated with TGFβ1 and DAPT or DMSO. Scale bars: 20 μm. (b) Quantification of the percentage of cells with nuclear MRTF-A as a function of cell seeding density. (c) Fluorescence microscopy images of MRTF-A localization in NMuMG cells cultured on micropatterned triangular and bowtie shaped islands of area 1600 μm^2/cell treated with TGFβ1 and DAPT or DMSO. Dotted white lines outline individual cells. Scale bars: 20 μm. (d) Quantification of the percentage of cells with nuclear MRTF-A as a function of the number contacts with neighboring cells.

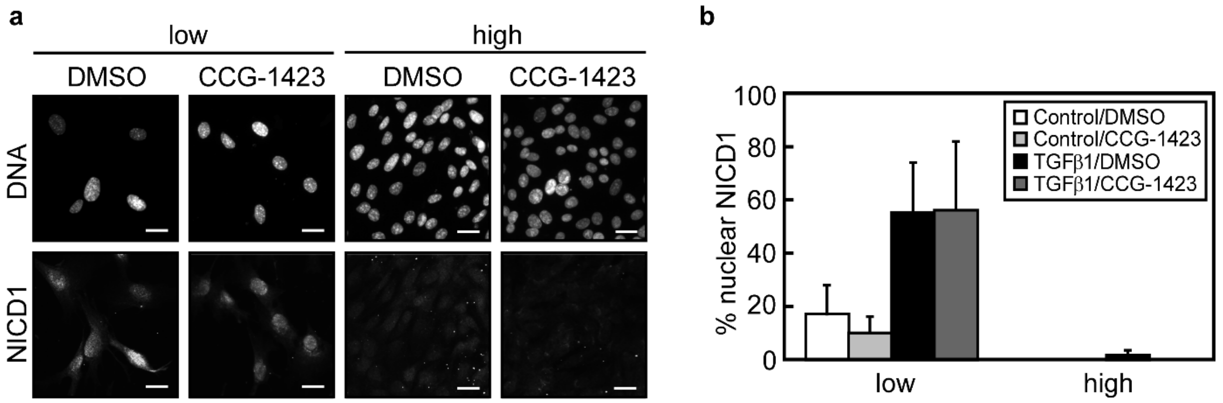

Figure S6. Inhibition of MRTF-A nuclear import does not impact Notch activation. (a) Fluorescence microscopy images of NICD1 localization in NMuMG cells seeded at low and high densities and treated with TGFβ1 and CCG-1423 or DMSO. Scale bars: 20 μm. (b) Quantification of the percentage of cells with nuclear NICD1 as a function of cell seeding density.
